# Vest Pocket Anatomist

**Published:** 1878-06

**Authors:** 


*The Vest-Pocket Anatomist*, (founded upon "Gray.") By C. HENRI LEONARD, A. M., M. D. Detroit: 1878. Price, paper, 50 cents.

It contains each bone, its name, points of interest, and muscles attached; each muscle, name, origin, insertion, nervous supply and action; each artery, name, course, structures supplied, and branches; each vein, name, course, where emptying, vessels received and number of valves; and each nerve, name, origin, course and distribution.

It is used in nearly all the Medical Colleges, and is of the greatest value to the student as a means of refreshing his knowledge. 50,000 copies have been ordered by a London firm for use in England.

---

We shall publish in our next an able article upon *Urea*, and the *Methods Employed* for its *Quantitative Determination*, by Prof TUCKER, of the Albany Medical College; and also a synopsis of the Proceedings of the next meeting of the American Medical Association.

